# Evaluation of the Mechanical Stability of Optical Payloads for Remote Sensing Satellites Based on Analysis and Testing Results

**DOI:** 10.3390/s25216546

**Published:** 2025-10-24

**Authors:** Dulat Akzhigitov, Berik Zhumazhanov, Aigul Kulakayeva, Beksultan Zhumazhanov, Alikhan Kapar

**Affiliations:** 1 Ghalam LLP, Astana Z05G9X0, Kazakhstan; 2 Department of Radiotechnics, Electronics and Telecommunications, International Information Technology University, Almaty A15M0F0, Kazakhstan

**Keywords:** payload, nanosatellite, FEM, vibration testing, damping coefficient, satellite

## Abstract

**Highlights:**

**What are the main findings?**

- Finite element analysis indicated sufficient stiffness of the optical payload, while vibration testing identified key modal frequencies within the 300–1340 Hz range, predominantly in the secondary mirror bracket and electronics unit.
- Discrepancies between simulation and test results highlighted the heterogeneous nature of damping, necessitating the incorporation of localized damping coefficients for improved model accuracy and reliable structural predictions.

**What is the implication of the main finding?**

- The identified modal frequencies and the heterogeneous damping characteristics necessitate a refined approach to structural modeling, moving beyond uniform damping assumptions.
- Accurate representation of these parameters in future designs is crucial for predicting payload response to launch loads and ensuring long-term operational reliability.

**Abstract:**

This paper presents the results of numerical modeling and vibration testing of a nanosatellite’s optical payload, aimed at assessing its mechanical stability under the mechanical impacts of launch. The purpose of the study is to compare finite element modeling (FEM) data with experimental testing to refine the computational model and improve the reliability of mechanical stability predictions. The methodology included an FEM analysis with an average damping coefficient, an adapter blank test, a resonance study with a low-level sinusoidal run, random vibration tests, and a sinusoidal pulse test. The FEM results showed an average yield margin of safety MoS = 2.5 with a minimum MoS = 1.8 in the primary mirror mount area. The adapter blank test confirmed the absence of natural resonances in the operating range. The resonance study revealed modes in the 300–1340 Hz range, with the most pronounced peaks in the secondary mirror bracket (520–600 Hz) and the electronics unit (1030–1100 Hz). A comparison of the root mean square (RMS) acceleration values between calculations and tests revealed discrepancies due to the heterogeneous nature of the damping. The values of ζ determined by the half-power method varied from 0.9% to 4.8%, which confirms the dependence of the damping properties on the frequency and localization of the modes. The obtained results confirmed the structural integrity of the payload, allowed for the localization of structural elements, and substantiated the need to consider actual damping coefficients in FEM models. The presented data can be used to optimize the design and improve mechanical stability during payload integration into the satellite platform.

## 1. Introduction

In recent years, the use of nanosatellites in Earth remote sensing (ERS) has grown rapidly, with optical payloads playing a crucial role in ensuring high-precision and high-quality imagery. Such systems face significant mechanical stress during satellite launch and in the harsh space environment, which can lead to unwanted deformations and defocusing of optical elements. Loss of focus or changes in system geometry significantly degrade image quality, which is critical for ERS applications. Therefore, reliable methods for assessing the stability of optical systems are needed to proactively identify and prevent potential problems. An integrated approach combining analytical modeling and in-kind vibration measurements is a relevant and sought-after solution, ensuring accurate predictions and stable operation of satellite optical systems under real-world operating conditions.

As noted earlier, nanosatellites have made significant progress in the field of remote sensing in recent years. For example, ref. [1] demonstrated that a combination of automatic image segmentation with GIS analysis and expert interpretation allows for high accuracy (approximately 83%) in mapping large areas (using the state of Michoacan in Mexico as an example). Nanosatellite constellations are also being actively developed; for example, ref. [2] demonstrated that NDVI calculated using Dove data can achieve high correlation with Landsat-8/MODIS (r^2^ ≈ 0.97–0.99), which ensures reliable monitoring of intra-field variability. These results are also confirmed by a meta-analysis [3], which emphasizes the rapid growth in the number of studies devoted to the agricultural application of remote sensing and the steady demand for data with high spatial and temporal resolution.

Along with agricultural applications, CubeSat constellations have also found application in hydrology. For example, in [4], PlanetScope data was first used to monitor the dynamics of lakes and rivers in Alaska, demonstrating the feasibility of reliably analyzing seasonal changes with an accuracy of up to 11% when validated against ultra-high-resolution images. Thus, modern nanosatellites have proven their effectiveness for agricultural and environmental applications, which creates a demand for further improvements in the quality of observations. In particular, the authors of [5] proposed an image-based strategy, adaptive staring attitude control, which compensates for camera calibration uncertainties and enables stable observation of dynamic targets.

These challenges cannot be addressed without increasing the engineering maturity of the platforms themselves. In recent years, CubeSat satellites have evolved from educational projects to reliable and standardized systems. Modular software frameworks, examples of successful missions with demanding optical payloads, and standardized hardware components ensuring safety and reliability during orbital launches have emerged [6,7,8].

Engineering studies also note that various telescope designs (refractor, reflector, and catadioptric) are used for CubeSats, and the choice of detectors (CCD or CMOS) is determined by the balance between sensitivity and limitations on mass and power consumption. Modern missions demonstrate the achievement of spatial resolution at the level of 1.5–4 m from orbit while maintaining the compactness of the system [9]. The application of similar principles is also confirmed in CubeSat missions. For example, the MeznSat (3U) project implemented a combination of a compact spectrometer for the shortwave infrared region and an RGB camera, which made it possible to solve problems of monitoring greenhouse gases and aquatic ecosystems with a power consumption of less than 1 W and a sensor weight of about 230 g [10].

Along with the development of optical systems, optical, thermal, and mechanical effects are also gaining particular importance. Reviews show that microvibrations with frequencies below 30–100 Hz and temperature gradients within the orbital cycle range can cause optics deformation and degrade image quality. Damping devices, low-expansion materials, and athermalization methods are used to compensate for these effects. However, for CubeSats, such approaches are currently being implemented on a case-by-case basis [11].

The importance of these factors is confirmed by practice; without full-scale thermal vacuum tests, it is impossible to guarantee the stability of nanosatellites in LEO conditions. Thus, in the EIRSAT-1 (2U) mission, temperature cycles from −16 °C to +56 °C were carried out in the ESA CSF in a vacuum of 10^−6^ mbar, which made it possible to identify hidden inconsistencies in the operation of the payload and adjust the battery charging profile [12]. Similar experience was obtained on 3Cat-4 (1U), where thermal modeling using Thermal Desktop was compared with the thermal-vacuum campaign in ESA’s ESEC-GALAXIA. The agreement between the calculated and experimental data within ±6 °C confirmed the correctness of the models and allowed for the optimization of heating and heat removal strategies [13]. These results emphasize that the combination of modeling and testing is a prerequisite for ensuring the thermomechanical reliability of CubeSats.

Along with thermal vacuum testing, mechanical components and retention systems are crucial for the reliability of nanosatellites. A number of missions employ solutions based on burn-wire mechanisms, initially developed in CanSat-level prototypes, which enable simple and predictable release of components with minimal mass [14]. More complex platforms emphasize damping and vibration resistance. For example, testing of deployable panels has shown that the use of damping inserts and optimized retainers reduces vibration loads (Grms) and ensures stable structural behavior during launches and orbital operation [15]. Such examples illustrate that even miniature mechanical components require careful design and qualification, since their failure can lead to the complete loss of payload functionality.

A similar approach is applied to other CubeSat functional modules. For example, for 50 mN-class microthrusters, it has been shown that the choice of housing material directly impacts thermal losses and thrust degradation. The difference between the “best” and “worst” materials reaches 4–7% [16]. Similar methods have been implemented in the development of microelectromechanical systems for thrust modules, where the integration of igniter, heater, and temperature sensor functions has minimized power consumption and ensured thermal stability even in harsh orbital conditions. Tests have confirmed the retention of characteristics after vibration loads and the effectiveness of the integrated thermal control [17]. Such examples demonstrate that the FEM and thermal-vacuum methods, successfully applied to microthrusters and mini-modules, are universal and can be used to improve the reliability of CubeSat optical systems.

Experience from large missions emphasizes that ensuring thermal stability is essential for the successful operation of optical instruments. Sentinel-3, for example, utilizes a comprehensive thermal architecture. The passive thermal control system, supplemented by heaters, multilayer thermal insulation, and radiators, features dozens of heating lines (35 primary, 35 backup), triple redundancy of temperature sensors, and more than a hundred thermistors for monitoring [18]. This design maintains stability within ±1 °C while requiring an accuracy of ±0.3 K for the SLSTR radiometer. For small platforms, such energy-intensive solutions are excessive in terms of weight and power consumption, so the emphasis is shifting to compact and energy-efficient methods. On the OroraTech mission (3U CubeSat), an ATP12 paraffin-based phased thermal accumulator was used for the IR camera. For an additively manufactured aluminum container with a capacity of ≈670 g, passive thermal stabilization solutions allowed the temperature fluctuation amplitude to be reduced from ~23.6 K to ~7.4 K per orbit and to ~1.6 K in active shooting mode, satisfying the ≤10 K requirement [19].

Also, after demonstrating that passive thermal stabilization solutions and proven FEM/ thermal vacuum approaches are scalable from optics to other mini-modules, it is logical to move on to empirical validation on real payload samples [16,19].

First, image quality retention has been demonstrated using “classical” optical systems, thanks to precise mechanics and thermal stability. The flexure-based autofocus mechanism ensures submicron positioning of the secondary mirror and limits degradation of the Modulation Transfer function MTF under vibration loads to ≤5% [20]. A more powerful 12U lidar configuration incorporates comprehensive vibration and thermal control measures (shock absorbers, heat pipes, thermoelectric coolers, and radiators), confirmed by modal and thermal calculations and compliance with GEVS requirements [21]. It has also been shown that lightweight modules, with proper integration of thermal control and monitoring, can withstand a full profile of vibration and thermal tests and operate stably in flight, including optical monitoring of biological objects [22].

Secondly, quantum payloads demonstrate a “stress test” for the most sensitive systems. On the 3U SpooQy-1 mission, an isostatically mounted source of entangled photons maintained alignment after vibration and thermal-vacuum tests and in orbit, which is confirmed by measured values of CHSH ≈ 2.60–2.63 [23], and a system analysis of SatQKD shows that the performance factors are optical losses of the channel and background suppression, with a loss reduction of just 3 dB increasing the annual secretion yield severalfold, especially in the case of BB84 protocols with decoy states [24,25].

Thus, global experience demonstrates that even the most sensitive optical and quantum payloads can maintain alignment and functionality, provided that their thermomechanical protection and comprehensive validation are carefully designed. However, for remote sensing satellites, ensuring the mechanical stability of optical systems under launch loads remains an important task. Structural dynamics and microvibration compensation are the focus of recent research.

Recently, considerable attention has been devoted to studying the dynamics of satellite structures with rotating or flexible elements, where vibrations and rotational irregularities affect the accuracy of attitude control and observations. In particular, refs. [26,27] present refined models of rotating payload dynamics that take into account active magnetic damping and centrifugal hardening of the structures, which can improve system stability and accuracy.

Finite element numerical methods (FEMs) are traditionally used to predict structural behavior. However, the use of averaged parameters and simplified boundary conditions does not always accurately reproduce the actual dynamic response of a system. This results in discrepancies between calculated and experimental data, reducing the reliability of predictions and complicating the assessment of the reliability of the payload.

Based on this, the objective of this study is to comprehensively assess the mechanical stability of the developed optical payload by comparing the results of finite element modeling and vibration testing. Particular attention is paid to verifying the computational model, analyzing resonance characteristics, and identifying the most vulnerable structural elements. This approach allows for more accurate prediction of the optical payload’s behavior under launch conditions and ensures more reliable integration into nanosatellite platforms.

## 2. Materials and Methods

This study examines the mechanical stability of the developed optical payload designed for a nano-class satellite platform. The work covers the stages of the payload’s mechanical integration and vibration testing aimed at assessing its strength characteristics. The study was conducted at Ghalam LLP, located in Astana, a leading organization in the space industry of the Republic of Kazakhstan specializing in the development, production, and testing of satellites, as well as scientific research in space technology.

### 2.1. Description of the Payload Design

**Figure 1:**
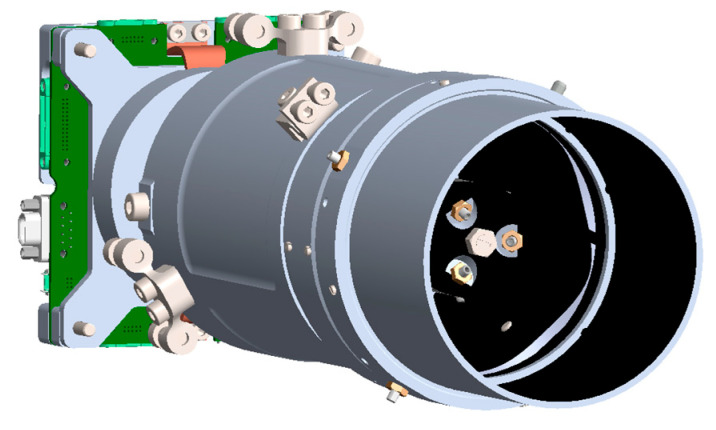
Model of optical payload in Solidworks CAD.

The developed payload is a compact mirror-lens system with an integrated detector unit and electronic board, which is shown in Figure 1. The geometry of the structure was designed using the Solidworks 2025 computer-aided design system.

The supporting structure is made of 7075 T6 aluminum alloy, providing a high strength-to-weight ratio. The optical elements, the primary and secondary mirrors, are made of 6061 T6 aluminum. The secondary mirror is mounted on a spider-type bracket and has six degrees of freedom, allowing for precise collimation. The primary mirror is rigidly mounted and integrated directly into the supporting structure.

The position of the optical components relative to the photodetector can be adjusted using movable adjustment elements, allowing for precise focal length adjustment. Once the alignment is complete and a clear image is obtained on the sensor, all adjustable elements are secured using liquid locking elements.

### 2.2. Integration of Payload

**Figure 2:**
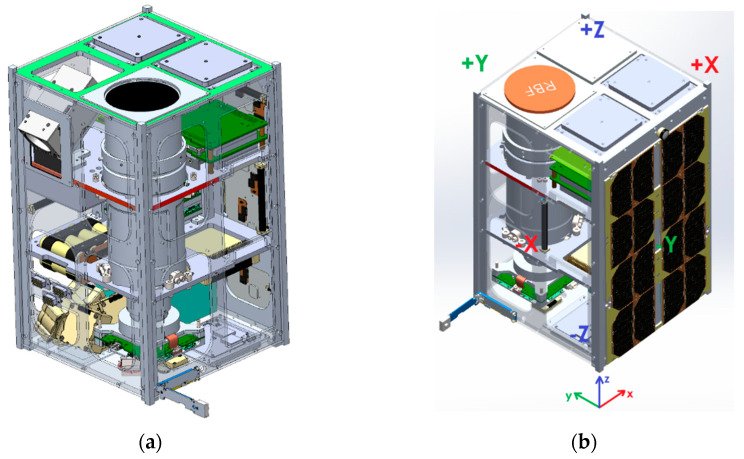
Accommodation of the payload inside the satellite: (**a**) Position of the payload inside 12U cubesat platform; (**b**) coordinate system for the platform.

The integration of the developed optical payload into the nano-class satellite platform is carried out in the configuration shown in Figure 2.

The payload is mounted on two transverse support plates of the platform’s internal structure. This mounting arrangement ensures the payload is firmly secured within the satellite’s body and aligns its optical axis along the +Z geometric axis. In the flight configuration, this axis is oriented toward the nadir, enabling imaging of the Earth’s surface during flight over the target area.

**Figure 3:**
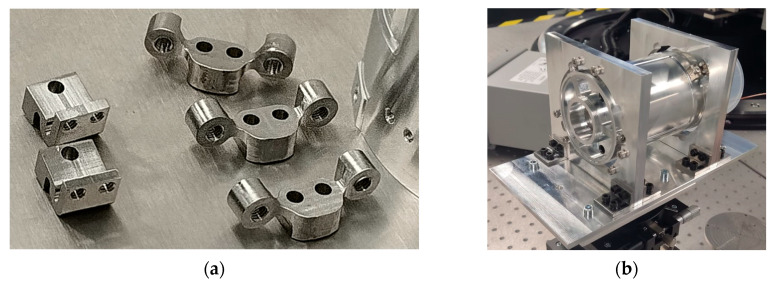
(**a**) Titanium fasteners; (**b**) the payload structure mounted in an adapter simulating the satellite platform.

The main mechanical elements for securing the payload are specially designed titanium brackets made of Ti6Al4V alloy (Figure 3). The combination of curved geometry and material properties—high rigidity, low thermal conductivity, and a low coefficient of thermal expansion—allows these fasteners to effectively isolate the payload from external thermal influences while ensuring secure attachment to the satellite platform.

Titanium fasteners are mounted to the payload housing and platform structure using DIN912 screws in sizes M3 and M4, with a recommended tightening torque of 2.2 Nm and 4.0 Nm, respectively. To prevent loosening of threaded connections, Scotch-Weld 2216 epoxy locking adhesive (3M, USA) is recommended.

### 2.3. Finite Element Model

To ensure the structural strength of the PN and to comply with radical deformations, a sequential analysis was performed using the finite element method (FEM) in the Ansys 2025 R2 software environment. The analysis included three stages: modal analysis, determination of natural frequencies, and calculation of the response to random vibrations.

The following conditions were adopted as strength criteria:

- All deformations must remain within the elastic range of materials;
- The minimum safety factor for all structural elements must exceed 2.0 throughout the entire period of load application.

**Figure 4:**
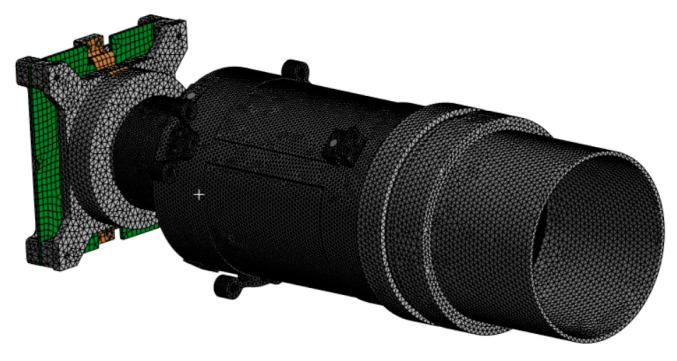
FEM mesh of payload containing 757,358 finite elements.

To adequately approximate the geometry and internal interactions of the payload, the model under study was decomposed into finite elements. Three-dimensional elements of the TETRA10 type were used in the calculations. The total mesh size was 757,358 finite elements (Figure 4).

**Figure 5:**
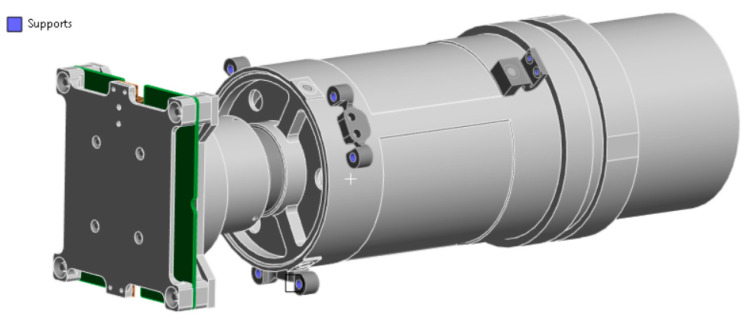
Payload supports in FEM simulation.

The analysis adopted a rigid connection to the platform using titanium fasteners as supports for the mechanical load (Figure 5). The bolted connections are simplified using a beam representation (1D beam) made of stainless steel, with deformable connections along the surfaces pressed by bolted connections (deformable/rbe 3). To reduce the computational costs of simulations, parts not involved in load transfer were geometrically simplified. In some cylindrical parts, radial contact was established “no separation”. In small parts integrated with the help of clamps, a rigid contact “bonded” was established.

Thus, the model takes into account both the design features of the fastenings and the realistic behavior of the contact surfaces, which ensures a correct assessment of the mechanical stability of the payload under external loads.

The material properties of the main structural components used in the FEM simulations are summarized in Table 1.

### 2.4. Mechanical Loads

The mechanical stability analysis of the payload included quasi-static calculations, modal simulations, and an assessment of the structure’s behavior under random vibration. The calculations were performed in accordance with the requirements of the SpaceX Rideshare program, as well as [28,29] standards. In accordance with these standards, design factors for various load directions were determined and used in the calculations. The values of the factors are presented in Table 2.

**Figure 6:**
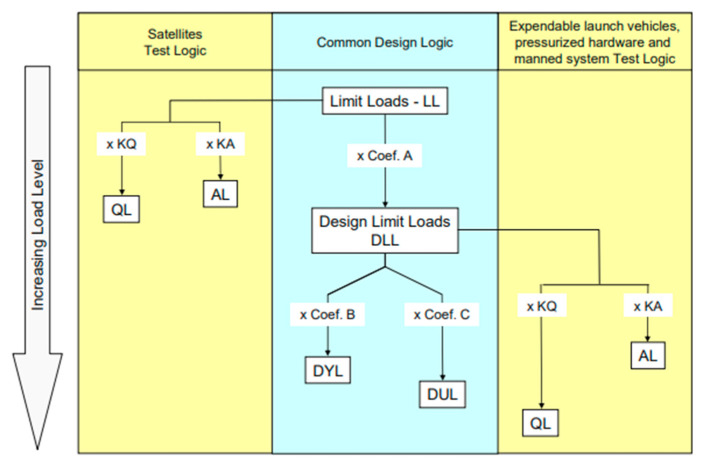
Design approaches from ECSS-E-ST-32-10 C 2009.

The calculation methodology used an approach to determining design loads that corresponds to the structure set out in [29]. Figure 6 shows the diagram for the formation of the LL limit loads. (Limit Loads) and DLL design loads (Design Limit Loads) depending on the test logic applied, for satellites, expendable launch vehicles, or manned systems.

In addition, this work used satellite test logic (Satellite Test Logic), in which the ultimate loads LL are multiplied by design factors depending on the direction and conditions of testing (e.g., KQ, KA), and form values for qualification loads (QLs) and acceptance loads (ALs).

Based on these data, a quasi-static test scenario for the payload was developed, and the corresponding values were used as input in the FEM simulations. This approach ensures compliance with international requirements for the design strength of a satellite.

**Figure 7:**
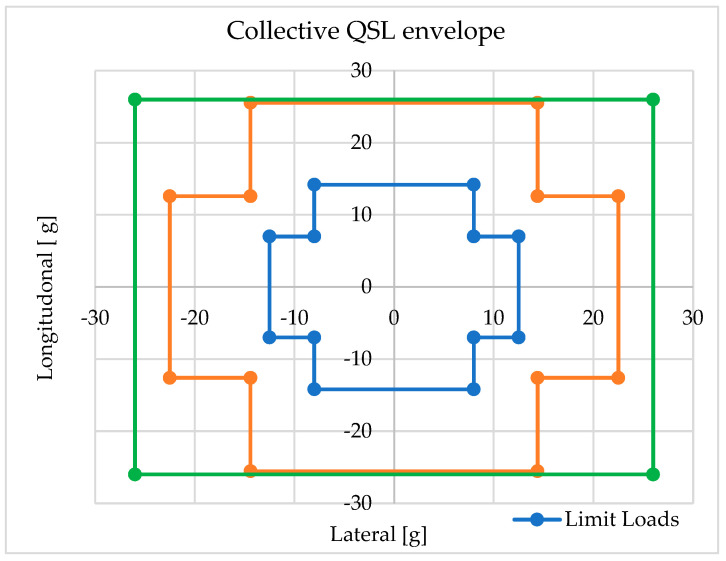
QSL profile of the payload.

Quasi-static load tests are conducted to verify the structural integrity of the primary components of a satellite platform during launch in a launch vehicle. During the preliminary design of a satellite platform, quasi-static load factors (QSLs) specified in the launch vehicle user manual are typically taken into account. QSLs represent more critical combinations of dynamic and static accelerations that may be encountered at any point during a mission, including ground and flight operations. Quasi-static load scenarios for payloads are based on QSLs. Falcon 9, multiplied by the design factor and then rounded, as shown in green in Figure 7.

The vibrational nature of the system is determined by the results of a modal analysis, which reveals the system’s natural oscillations, i.e., the patterns of system motion that arise at its natural frequencies. The result of this analysis is a set of frequencies at which resonance can occur. The system’s stiffness and damping are designed to achieve the first natural frequency above the launch vehicle’s vibration range or to prevent natural frequencies from occurring at peak system vibrations. Natural frequencies ranged from 20 to 2000 Hz.

The modal analysis results are then used to calculate the loads arising from random launch vehicle vibrations, determined by the power spectral density (PSD). As a result, the maximum stress values from the obtained statistical data are determined by a normal distribution with a standard deviation of 3σ, meaning that the instantaneous acceleration of the system due to vibrations will lie within the obtained values in 99.73% of cases. The specified PSD values for the frequency ranges were used as input parameters in the FEM calculations and are presented in Table 3.

**Figure 8:**
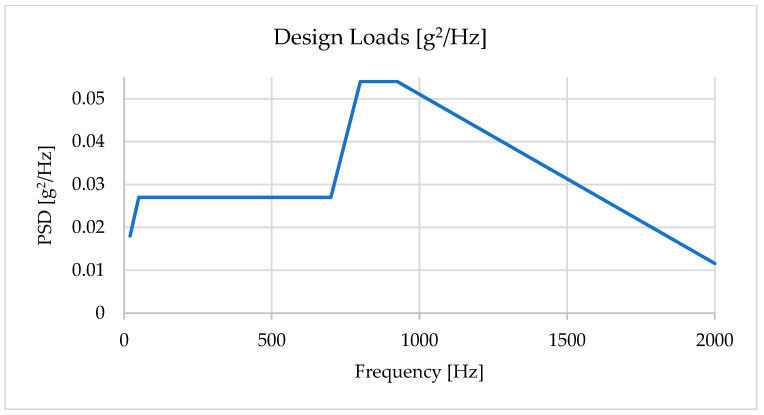
PSD design profile based on Falcon 9 data.

For clarity, the resulting PSD profile is presented as a dependence of spectral density on frequency (Figure 8). This graph was used to verify input loads during simulations.

Thus, the PSD design profile based on Falcon 9 data was adopted as an input for the FEM simulations and subsequent vibration tests of the payload, which ensured correct modeling of real launch conditions.

### 2.5. Vibration Test Procedure

Vibration tests were aimed at verifying the strength and quality of the payload assembly, determining resonant frequencies, and the structure’s response to mechanical stress. To simulate loads on the payload inside the platform, the payload must be secured to the sliding table using a transition structure that does not generate additional loads through its own resonant effects.

**Figure 9:**
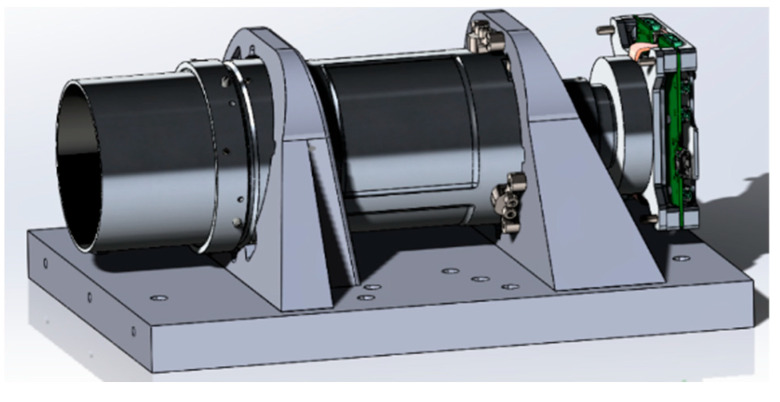
Payload model in an adapter for vibration testing.

For these reasons, a rigid steel structure (adapter) was developed for vibration testing. It was designed to connect the payload to the sliding table and simulate the conditions of the payload mounting in the satellite. The adapter consists of a 34.3 cm × 22.4 cm plate and two flanges for supporting the payload in a horizontal position, secured to the plate with M8x16 bolts. The total weight of the adapter was approximately 20.2 kg. A model of the payload in the vibration testing adapter is shown in Figure 9.

**Figure 10:**
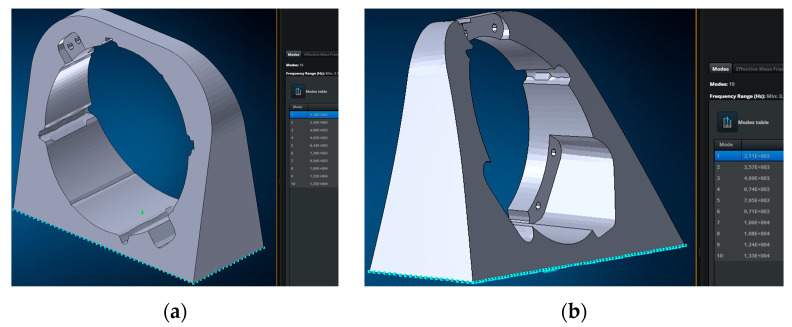
Natural frequencies of the adapter flanges: (**a**) flange 1 with first natural frequency equal to 2160 Hz; (**b**) flange 2 with first natural frequency equal to 3110 Hz.

**Figure 11:**
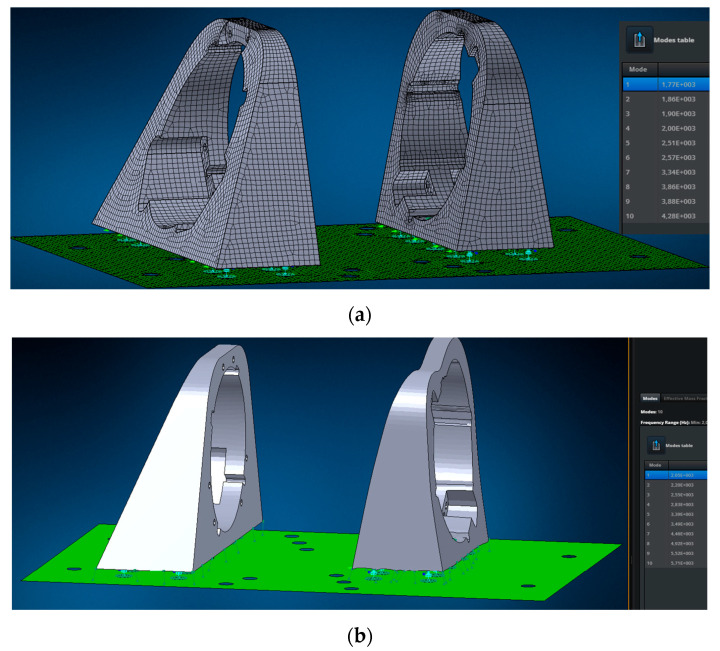
Modal analysis of the adapter: (**a**) with only bolt connections holding the flanges to the plate; (**b**) with bolt connections and additional connections representing contact between the flanges and the plate.

This adapter was designed with resonant frequencies above approximately 2000 Hz. This condition avoids additional loads on the payload during testing and maintains the objectivity of the results. This condition was verified using modal analysis in Nastran 2023.4 software (Figure 10 and Figure 11).

As can be seen from the results, the flange on the left has the first natural frequency at 2160 Hz, the flange on the right at 3110 Hz, i.e., the flanges are suitable for the test according to the stiffness requirements.

The flanges are attached to the plate only by means of rigid fasteners RBE 2, simulating bolted connections, yielding a first natural frequency of 1770 Hz (Figure 11a). The fastening rigidity is increased by additional connections to the 2D plate; all natural frequencies exceed 2000 Hz (Figure 11b).

**Figure 12:**
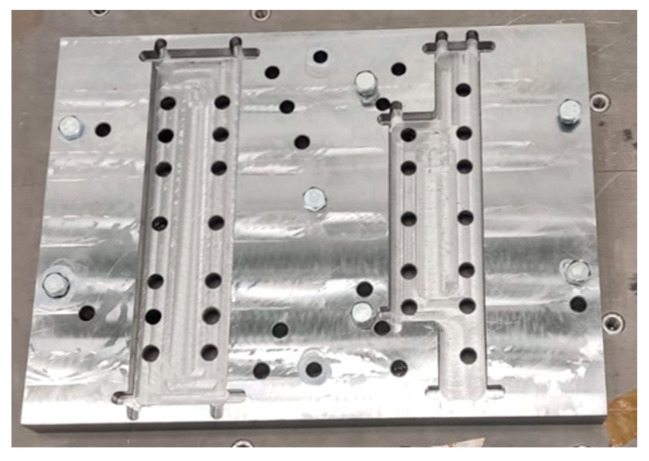
Adapter plate for vibration testing.

Since the adapter is an assembly design, each component was engineered to achieve sufficient rigidity. A key design consideration to avoid adapter natural frequencies below 2000 Hz was ensuring high-quality fastening of the flanges to the adapter plate. Therefore, it was decided to fabricate the plate with cutouts for the flange ends. The adapter plate for vibration testing is shown in Figure 12.

To confirm the design results of a vibration adapter, a blank test of the adapter—a low-amplitude sinusoidal test without mounting the payload—should be performed before the main vibration tests. The purpose of this test is to establish resonant vibrations within the required frequency range to minimize external influences on the payload during the test.

Vibration tests were conducted at Ghalam LLP’s TestLab test site on a V 8-440 vibration rig (LDS Test and Measurement Ltd., UK) using single- and three-axis accelerometers. The tests were conducted in ISO 8 cleanroom conditions, under atmospheric pressure, and at 22–24 °C temperature. During assembly, the mechanical parts of the payload and the adapter were checked for geometric tolerances under similar temperatures to reduce thermomechanical effects. The tests included the following tests on all three axes:

- Low-amplitude sinusoidal resonance examinations;
- Random vibration tests according to Falcon 9 User’s Guide;
- Sinusoidal impulse tests (shock test).

**Figure 13:**
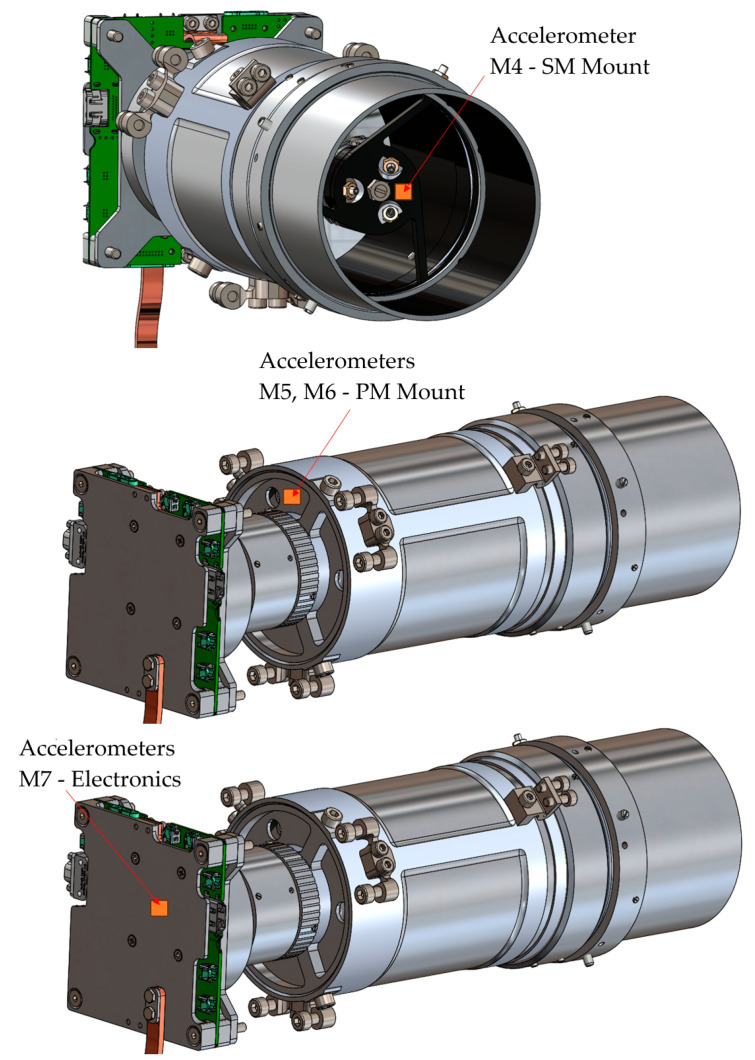
Diagram of accelerometer mounting locations. 1. Accelerometer 1 (PM Mount) is mounted near the primary mirror mount and records the vibration response in the area of the support structure. 2. Accelerometer 2 (SM Mount) is mounted on the secondary mirror bracket to control the stability of the optical unit. 3. Accelerometer 3 (IAS) is placed on the interface mounting plate, providing registration of the transmitted loads to the entire payload system.

The random vibration load exceeded the flight load by 20%. The payload assembly was carried out according to the procedure applied during the assembly and integration of the satellite flight model. Piezoelectric accelerometers (Brüel & Kjaer, UK) were used to record the payload structure’s response during vibration testing. A total of three sensors were installed, the locations of which are shown in Figure 13.

This distribution of sensors allows monitoring both global responses of the housing and local vibrations of critical optical elements, which provides a comprehensive assessment of the dynamic stability of the payload.

Before conducting the main random vibration tests, a resonance survey of the payload was performed. The purpose of this test was to determine the structure’s natural frequencies, as well as to verify the correct installation of the accelerometers and the reliability of the fasteners.

Tests were conducted in the frequency range from 5 to 2000 Hz and at a sweep rate of 1 octave/minute in three directions (X, Y, Z). The test parameters are presented in Table 4.

**Figure 14:**
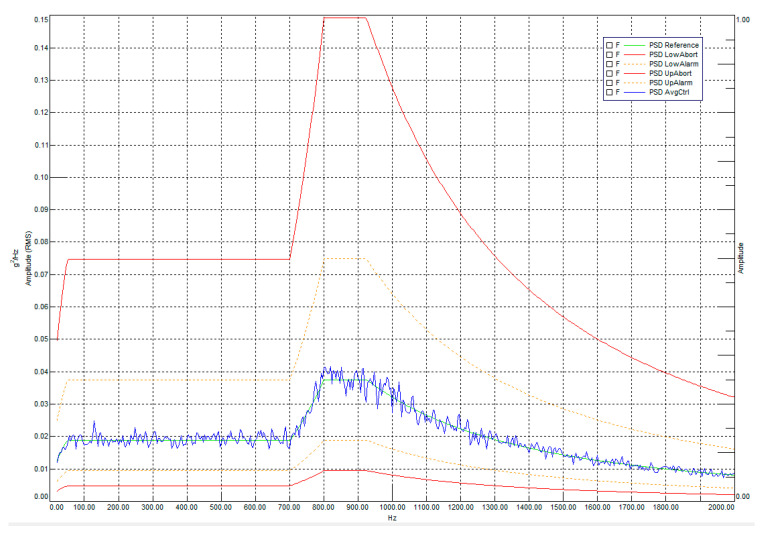
Input PSD for random vibration testing.

Figure 14 shows the input power spectral density (PSD) profile used to verify the frequency range and correct excitation.

The control accelerometer (Brüel & Kjaer, UK) was configured to warn when acceleration exceeds +3 dB from the nominal level and automatically shut off when it exceeds +6 dB, eliminating the risk of damage to the structure.

In addition to the resonance survey, the payload was subjected to random vibration and sinusoidal pulse tests. The input test parameters were determined in accordance with [28,29,30].

### 2.6. Random Vibrations 

The input values of the power spectral density (PSD) used to simulate random vibrations in the frequency range of 20–2000 Hz are given in Table 5. The test duration was 2 min in each of the three directions (X, Y, Z), with an integral level of GRMS = 6.23

### 2.7. Sinusoidal Pulse

This test is designed to simulate overloads during stage separation and dynamic impacts. The test input parameters are listed in Table 6. The tests were performed at a frequency of 15 Hz with an amplitude of 12.5 g in three directions (X, Y, Z), with acceleration, loading, and deceleration phases. The initial data used for the sinusoidal pulse test are listed in Table 6.

Thus, the tests conducted made it possible to simulate real vibration effects typical for the launch conditions of a payload as part of the Falcon 9 launch vehicle.

## 3. Results

### 3.1. Finite Element Analysis

**Figure 15:**
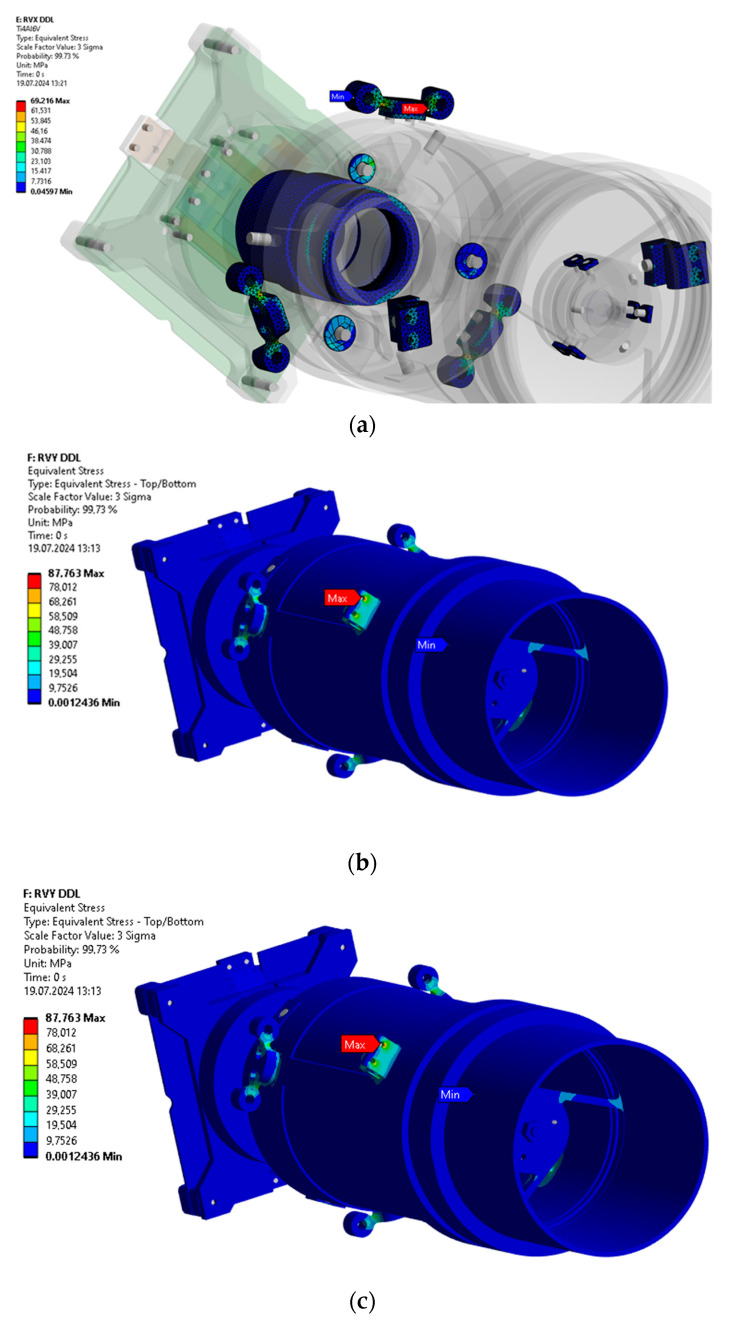
Examples of stress results in the von Mises payload structure along 3 axes: (**a**) Contour plot of components with highest equivalent von Mises stress with 3σ-probability during random vibration load along the main axis; (**b**,**c**) Contour plots of components with highest equivalent von Mises stress with 3σ-probability during random vibration load along lateral axes.

A standard deviation of 3σ shown in Figure 15 below demonstrates that the majority of the loads are concentrated in the payload carrier fasteners to the satellite platform. The obtained results were calculated for an average damping value of 2%.

The calculation of the safety margins for the yield strength in the materials of the structure was carried out, showing an average value of about 2.5. The lowest value of MoS was found at the primary mirror mount to the supporting structure, equal to 1.8, with random vibrations along the vertical Z axis.

(1) $$MoS=\frac{\mathrm{Admissable stress}}{\mathrm{FOS}\times \mathrm{Maximal stress in the structure}}-1$$

### 3.2. Vibration Test Adapter Test Form

**Figure 16:**
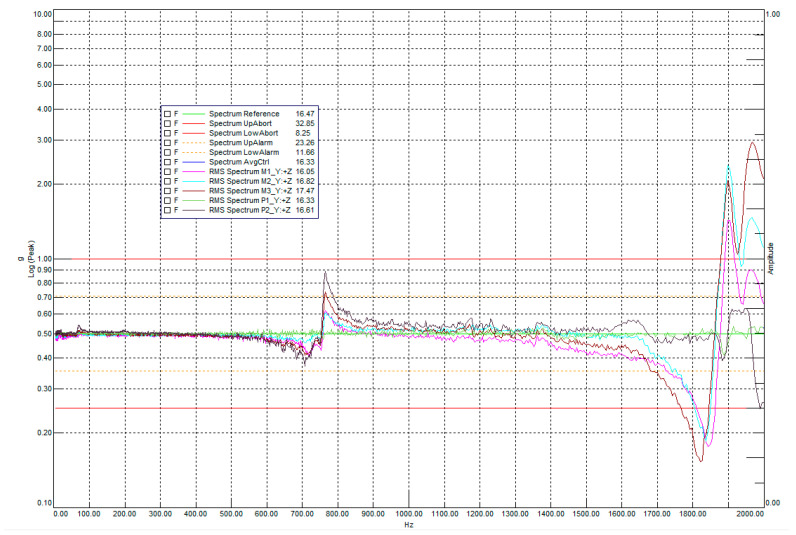
Graph of adapter acceleration during a 0.5 g sinusoidal blank test along the vertical Z axis.

To confirm the adapter’s rigidity and stability, a blank test was conducted. Resonance during the test is characterized by an increase in acceleration amplitude exceeding +6 dB. During the blank test, the adapter was also subjected to random vibrations and a sine-wave impact test to determine assembly quality. Figure 16 displays the acceleration of the adapter during a 0.5 g sinusoidal blank test along the Z-axis.

Oscillations around 750 Hz had a slight increase in acceleration amplitude (less than +3 dB); oscillations in the 1800–2000 Hz range were specific to the sliding table used for the test. Overall, the results show no adapter-induced resonances.

The amplitude-frequency response of the developed vibration adapter, obtained during a blank test, confirmed the absence of resonances in the required frequency range (see Figure 10 and Figure 11). This result demonstrated the adapter’s suitability for obtaining objective results of payload vibrations. Final assembly for testing was then performed, tightening the adapter bolts to 80% of the yield strength and using Lactite and 3M DP109 liquid fasteners, and preparing the equipment for testing.

### 3.3. Vibration Tests of Payload

Vibration qualification tests of the optical payload were carried out to achieve the following objectives:

1. Checking the structural strength and quality of the payload assembly.
2. Determination of resonant frequencies for comparison with the analysis results.
3. Determination of the structure’s response to random vibrations in critical locations and further correlation with the analysis.
4. Confirmation of the optical system’s stability under mechanical loads.

The tests will include resonant frequency tests (low-level sine sweep), random vibration tests, and sine wave tests (sine burst). All types of tests should be carried out in turn with the fasteners corresponding to the loads in Z, then X and Y, in the following sequence:

1. Resonant test 1 (low frequency level).
2. Random vibrations.
3. Resonant test 2 (low frequency level).
4. Sinusoidal pulse.
5. Resonant test 3 (low frequency level).

An important criterion was the shift in resonance frequencies after random vibration and sinusoidal pulse tests relative to the previous resonance test. Resonance is characterized by an increase in acceleration amplitude exceeding +6 dB. This shift, exceeding 5%, may indicate a defect, such as a loose bolt or structural damage.

**Figure 17:**
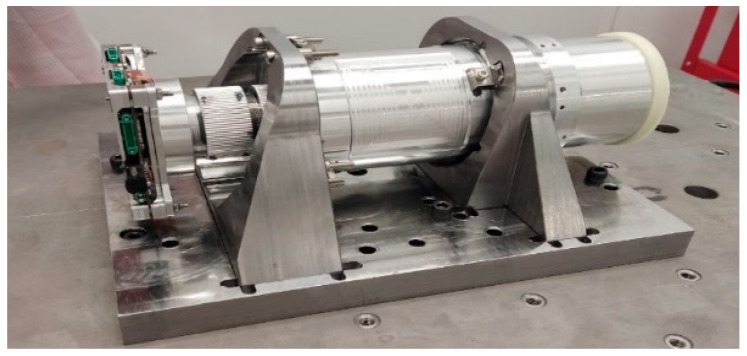
The payload mounted on a vibration adapter.

The payload was secured in a specialized adapter that simulated the mounting conditions as part of the satellite platform, which is shown in Figure 17.

The result of the preparation was the reliable fastening of the payload in the adapter, which ensured the reproduction of the conditions of real fastening as part of the satellite platform and made it possible to conduct tests over a full range of loads.

**Figure 18:**
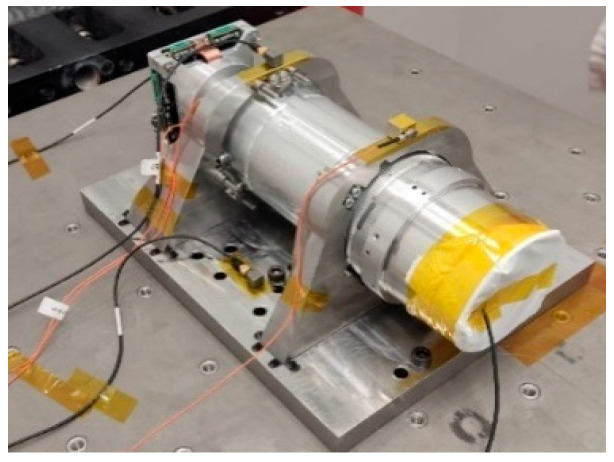
Payload with installed sensors.

Before testing, accelerometers were installed on the structure and placed at key points of the optical system, as shown in Figure 18.

Figure 18 shows the stage of preparing the measuring system. The installation of accelerometers allowed us to record the structure’s vibration responses in three directions (X, Y, Z) and compare them with the calculated FEM analysis data.

**Figure 19:**
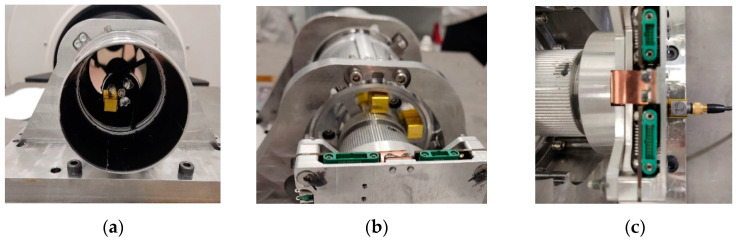
Photos of aluminium extenders wrapped in yellow kapton, which were used to mount the accelerometers. Accelerometer locations: (**a**) secondary mirror (M4); (**b**) primary mirror (M5, M6); (**c**) Electronics unit (M7).

The locations of the sensors are shown in Figure 19, which made it possible to monitor the response of critical elements of the payload.

This stage resulted in the identification of test points, the secondary mirror bracket (M4), the primary mirror support (M5, M6), and the electronics unit (M7). The data obtained allowed us to evaluate the behavior of the critical components of the payload and confirm the absence of changes in frequency characteristics after testing.

The results of the resonance survey of the payload revealed the presence of several vibration modes in the frequency range from 350 to 1180 Hz. The identified modes and corresponding vibration modes are presented in Table 7.

Analysis revealed that at low frequencies (350–630 Hz), localized vibrations of the electronics unit and the lower part of the structure were observed, while in the range of 730–855 Hz, vibrations and rocking movements of the secondary mirror bracket were detected. At higher frequencies (1030–1180 Hz), global vibrations of the entire structure and local resonances of the electronics unit were detected.

**Figure 20:**
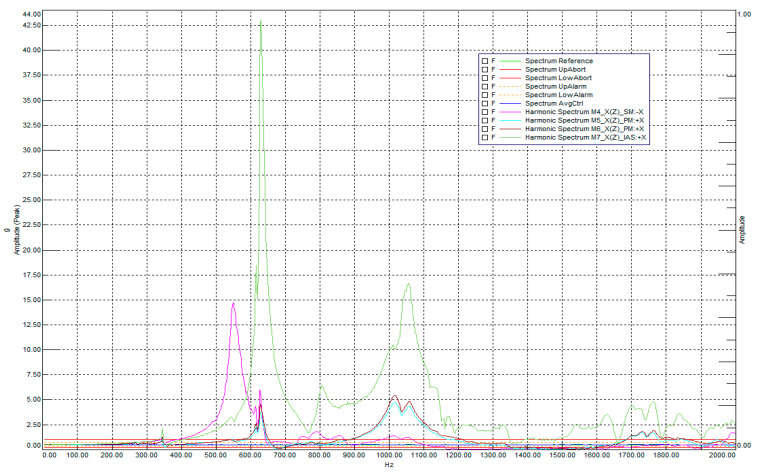
Resonance survey along the main optical axis (X-axis on the sliding table). The main peaks were observed in the secondary mirror bracket M4 (purple) and the electronics unit (green) M7.

Figure 20 shows the results of the resonance examination of the payload along the main optical axis (X-axis on the sliding table).

An analysis of the amplitude spectra revealed pronounced resonance peaks in the area of the secondary mirror bracket (sensor M4, purple curve) and the electronics unit (sensor M7, green curve). For the secondary mirror bracket, the maximum acceleration values were recorded in the range of 550–600 Hz, while for the electronics unit, around 1030–1090 Hz. The remaining sensors (M5, M6) recorded significantly smaller amplitudes, not exceeding the +6 dB threshold, which confirms the localized nature of the vibrations of these structural elements.

**Figure 21:**
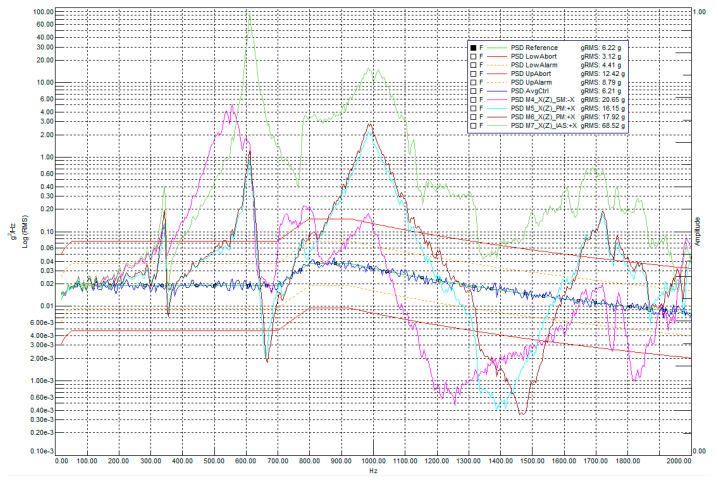
Logarithmic profile of loads from given random vibrations along the main optical axis.

Figure 21 shows the logarithmic spectral profile of the response to random vibrations along the main optical axis (X-axis).

The logarithmic graph in Figure 21 clearly demonstrates the amplitude-frequency behavior of the payload during random vibrations along the Falcon 9 profile. The PSD AvgCtrl signal indicates the acceleration values of the reference accelerometer mounted on the vibration adapter plate. This signal, compared to the target PSD Reference profile, as well as the RMS value of its acceleration (6.21 g compared to the input value of 6.22 g), demonstrates the controllability and accuracy of the test. Overall, the resonance profile during random vibrations is expectedly consistent with the results of the low-amplitude sine test, with a slight shift of the peaks closer to the center due to the statistically Gaussian nature of the vibrations. The main results of these measurements are the RMS acceleration values calculated from the integrals of the PSD profiles of the accelerometers, which provide data on the average vibration loads and the relative increases in vibrations measured in dB.

Below, Table 8, Table 9 and Table 10 present the main observations on the resonant frequencies of the payload along each axis. After each test, this amplitude-frequency characteristic was checked for frequency shift. The largest shift was detected in the second mode along the X-axis (electronics unit). This component was inspected for defects, none of which were found, which could indicate “settling” of the bolted connections in the numerous sandwich layers of this unit.

Using the results obtained from vibration tests, it is possible, through correlation with the simulation results, to adjust the dynamic FEM model for a more accurate further analysis. The key value for this is the damping coefficient ζ, which, as mentioned earlier, was assumed to have an average value of 2% over the entire structure in the finite element model. However, in reality, damping depends on many factors, especially the material and assembly method of the structure, and is a heterogeneous value. Based on the results of increasing accelerations, calculated from the ratios of the RMS values of random vibrations at the test output and input (Table 11), the difference in the resulting loads can be seen.

**Figure 22:**
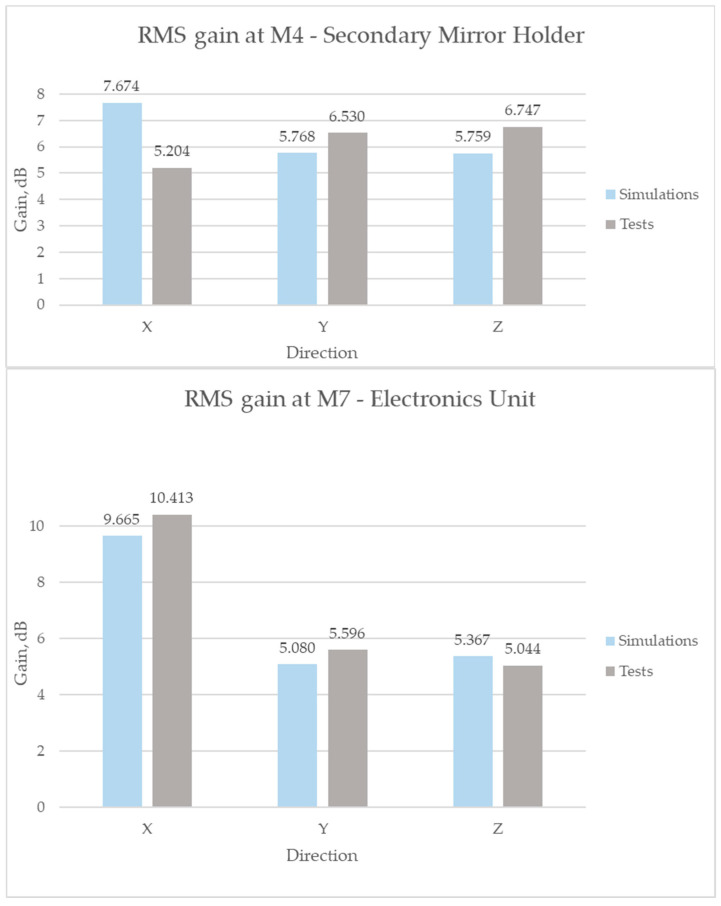
Comparison of RMS gains during random vibrations.

As shown in Figure 22**,** the RMS gains obtained from FEM simulations are in good agreement with the experimental results.

In most cases, a decrease in RMS values in a test relative to simulations indicates a larger change in ζ than predicted, and vice versa. This fact indicates the need to adjust the damping coefficient to improve the accuracy of dynamic simulations. Knowing the actual damping values is also important in relation to the damping quality factor (factor Q), defined as:

(2) $$Q=\frac{1}{2\zeta }$$

Mechanical systems are often designed to achieve 10 < Q < 50, i.e., 1% < ζ < 5%. The basis for this requirement is the fact that with insufficient damping, the system becomes excessively resonant, which is dangerous for the integrity of the structure, and with too much damping, the system becomes overdamped, which can lead to an increase in the time it takes for the system to reach equilibrium.

Determining the damping coefficient of a structure based on test results is possible using the half-power bandwidth method (half—power bandwidth). This is done by determining the frequency corresponding to the maximum acceleration of the structure on the axis of interest (with a pronounced response). Then, the frequencies of the half-power responses closest to this value are determined using the relationship described in Equation (1). The difference between these frequencies and the maximum response frequency is used to determine the damping ratio using the equation:

(3) $$R_{half-power}=\frac{R_{max}}{\sqrt{2}}$$

where R half-power is the acceleration at half power (g); R max is the maximum acceleration (g).

(4) $$\zeta =\frac{∆\omega }{2\omega_{n}}$$

where ζ is the damping coefficient, ∆ω is the difference between half-power frequencies (Hz), and ω_n_ is the frequency at R_max_.

Based on the half-power bandwidth method, the damping factors for the main peaks in the amplitude-frequency characteristic were calculated from the results of the low-amplitude sine wave test (Table 12)

As can be seen from the results, damping ζ is heterogeneous, depending on the frequency and localization of vibrations in the structure. To adjust the FEM mechanical model and improve the accuracy of the analysis, the current values of the damping coefficients should be taken into account. This can be accomplished in various ways. In particular, to identify the worst-case scenario for the structure’s behavior under external loads, it is advisable to use the minimum recorded value of ζ.

Furthermore, the equations and method of Reyle damping [31] can be used to approximate damping coefficients in subsequent stages of numerical modeling. At this stage, calculations using this method have not been performed; however, it is considered a promising approach for improving the accuracy of reproducing the dynamic response of a structure.

## 4. Discussion

Vibration tests provided data on the behavior of the optical payload under mechanical stress typical of the orbital insertion phase. According to the results, the greatest loads are in the direction of the primary optical axis X, due to the increased mass involved in vibrations, as well as the presence of layered “sandwich “ structures, which reduce overall rigidity. This load distribution should be considered when designing fasteners and ensuring the payload’s vibration resistance.

The modal analysis covered resonant frequencies up to 2000 Hz, which meets typical satellite requirements and ensures a sufficiently comprehensive assessment of the structure’s dynamic stability. The agreement between the identified vibration modes and the numerical simulation results demonstrates the validity of the computational model and its applicability during the design phase. The obtained data on the main resonant modes (Table 6) are of practical value for the subsequent integration of the payload into the satellite’s mechanical structure, particularly when matching the components’ natural frequencies to external dynamic influences.

When exposed to random vibrations of 20 gRMS, average acceleration values within the structure were within acceptable limits. However, peak accelerations of up to 69 g RMS were recorded in the area housing the electronics unit. This requires special attention in the further design of structural elements in this area, including possible reinforcement of support assemblies and cable connections.

Upon completion of the tests, a visual and functional inspection of the payload was conducted. No damage was detected, and functional checks confirmed full functionality. Therefore, it can be concluded that the payload successfully withstood the specified vibration load profile and demonstrates sufficient mechanical stability for subsequent qualification stages and integration into the satellite.

Also, the obtained experimental values of the damping coefficient ζ demonstrated pronounced heterogeneity, indicating the sensitivity of the structure’s damping properties to the frequency characteristics and localization of vibration modes. This heterogeneity complicates the application of uniform averaged parameters in finite element models and requires a more flexible approach to their approximation.

A comparative transmissibility analysis showed that the absence of damping leads to a 54% increase in transmitted forces in resonant modes, confirming the critical importance of external damping mechanisms in preventing structural failures [32].

Also, to improve the stability of optical systems under orbital microvibrations (microvibrations with frequencies below 30–100 Hz are critical), it was proposed to use a platform with an electromagnetic damper based on a parallel manipulator, the effectiveness of which was confirmed by simulations and experiments [33]. In parallel, sophisticated algorithms such as Model Predictive Control (MPC), which can account for the interaction of the control system and the flexible dynamics of the satellite, are being used as an advanced solution to ensure the pointing accuracy and stability of flexible systems (especially relevant for satellites with rotating sensors) [34].

Further enhancing the reliability of dynamic analysis requires considering the fundamental effects inherent in flexible structures subject to rotation. For example, for spacecraft with rotating payloads connected by a flexible boom, the effect of centrifugal hardening (stress stiffening) is important. Neglecting this effect, which depends on the rotation speed, leads to incorrect predictions and the risk of structural instability. Thus, accurate modeling of flexible dynamics, including nonlinear strengthening effects, is important to ensure the overall stability of the system [27].

One promising solution may be the use of the Rayleigh damping method, in which a symmetric damping matrix is formed as a linear combination of the mass and stiffness matrices. The equations presented in this paper demonstrate the mathematical basis for this approach and provide the ability to calculate the Rayleigh coefficients (α and β) based on the system’s modal parameters.

Although calculations using the Rayleigh method were not performed in the current study, its use could prove an effective tool in subsequent modeling stages. This is especially relevant when it comes to improving the accuracy of dynamic analysis and accounting for the impact of damping on the structural behavior under various loading scenarios. In particular, the Rayleigh approximation of ζ can be useful both for pessimistic (worst-case) estimates and for adaptively tuning model parameters depending on the frequency range of interest.

There are other aspects in addition to the heterogeneous nature of damping that may induce the deviations between numerical and experimental results. Firstly, considerations of finite element modelling, such as settings of contacts between closely fitting components, which are usually modeled as linear contacts to facilitate computations but may actually present more nonlinear behavior, inducing discrepancies. This is, for example, applicable to some fitting cylindrical components, which were fixed together at several points using glue; such connections were assumed as bonded but may in fact have had nonlinear contacts. Additionally, potential sources of errors may come from bolt preloads, which have some deviation in their coefficient of friction and inaccuracy in measurement by a torque wrench. Other sources of deviations mainly include manufacturing misalignments, which may reduce actual contact surface area between bodies, which is difficult to model in an FEM simulation; such discrepancies are usually managed by tight production control.

**Figure 23:**
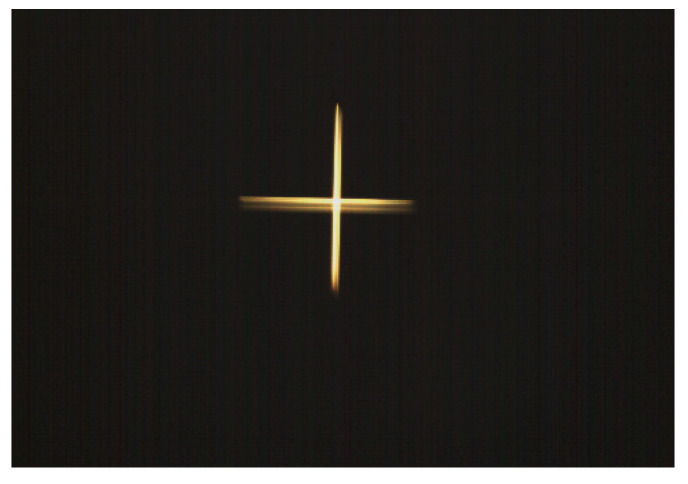
Target image acquired prior to vibration testing.

**Figure 24:**
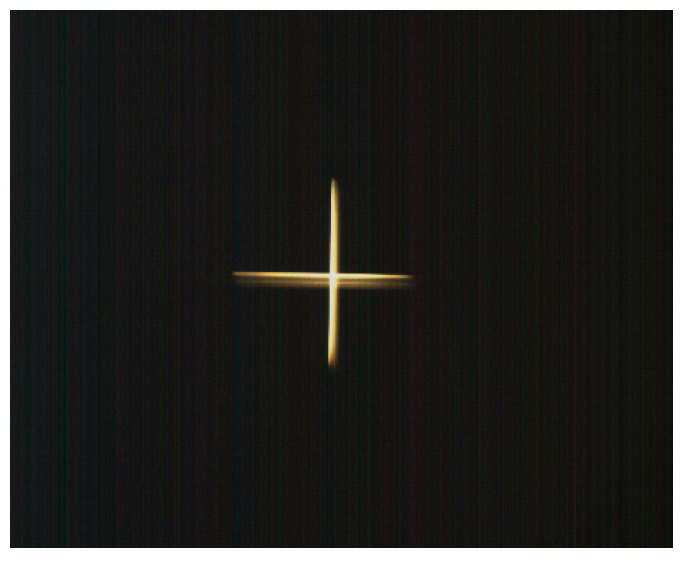
Target image acquired following vibration testing.

To assess the impact of vibration testing on image quality, benchmark images were acquired using an optical collimator with a focal length of 3 m. An initial image, obtained prior to vibration testing, is presented in Figure 23. Following the completion of vibration testing, a subsequent image was acquired using the same collimator under identical conditions. Visual comparison of the pre- and post-vibration images, as depicted in Figure 24, reveals no discernible degradation in image quality, thereby demonstrating the structural integrity and performance stability of the optical payload.

Thus, the results obtained in this study complement existing research based primarily on numerical analysis. For example, in [1], the thermomechanical stability of a compact optical payload was investigated using finite-element analysis, without subsequent experimental verification. Unlike that approach, this study incorporates both computational and in-kind methods for confirming the structural strength and thermal stability, allowing for a more reliable assessment of the system’s suitability for orbital operation.

## 5. Conclusions

Numerical simulation using the FEM method with an average damping coefficient of ζ = 2% demonstrated sufficient rigidity of the optical payload under typical launch loads. The average safety factor was approximately 2.5, with the minimum value of MoS = 1.8 recorded in the primary mirror mount area. To verify the correctness of the test bench, a blank test of the vibration adapter (0.5 g, Z-axis) was performed, which confirmed the absence of natural resonances in the operating range; the increase in amplitude around 750 Hz did not exceed +3 dB. Resonance testing revealed modes in the 300–1340 Hz range, with the most pronounced peaks recorded in the secondary mirror bracket (M4, 520–600 Hz) and the electronics unit (M7, 1030–1100 Hz), while for sensors M5–M6, the amplitudes did not exceed the +6 dB threshold. Comparison of RMS accelerations between simulation and testing showed discrepancies due to the heterogeneous nature of the damping, which was confirmed by additional half-power analysis. The ζ coefficient varied within the range of 0.9–4.8% depending on the frequency and localization of the modes.

Thus, the combination of numerical analysis and bench testing confirmed the structural integrity of the payload, identified zones with the highest concentration of dynamic loads (M4 and M7), and demonstrated the applicability of FEM for design calculations. The obtained results provide a comprehensive assessment of the mechanical stability of the optical payload, refine the parameters of the calculation models, and serve as the basis for developing measures to enhance mechanical stability and improve the reliability of the structure during integration into the satellite platform.

## 6. Patents

The patent for utility model № 9581 « Catadioptric lens of nano-class space satellites for remote sensing of the Earth» is one of the results from the work reported in this manuscript.

## Figures and Tables

**Table 1:** Material properties of main components for the FEM simulations.

Part Type	Material	E [GPa]	Rm [MPa]	Re/Rp_0.2_ [MPa]
Structure	7075 T6	73	554	494
Lens holder, Mounts	Ti6Al4V	106	918	846
Primary mirror	6061 T6	68	313	259
Fasteners	SS A2-70	195	700	450

**Table 2:** Design load factors.

Load Factors			
K_P_	K_M_	K_Q_	K_X_
1.2	1.2	1.25	1.8

**Table 3:** Initial PSD values for the random vibration design profile (Falcon 9).

**Frequency [Hz]**	20	50	700	800	925	2000	GRMS
**PSD [g^2^/Hz]**	0.018	0.027	0.027	0.054	0.054	0.011592	7.47

**Table 4:** Requirements for resonance examination.

Frequency Range (Hz)	Acceleration (g)	Scan Speed (oct/min)	Directions
5–2000	0.5	1	X, Y, Z

**Table 5:** Power spectral density settings for the vibration test.

Frequency (Hz)	20	50	700	800	925	2000	GRMS	Directional Axes	Duration (min)	GRMS
PSD (g^2^/Hz)	0.0125	0.01875	0.01875	0.0375	0.0375	0.00805	6.23	X, Y, Z	2	6.23

**Table 6:** Input data table for sine wave test.

Sine Test Settings/Sine Burst Settings	
Frequency (Hz)	15
Amplitude (g)	12.5
Acceleration periods	3
Load periods	5
Periods of slowdown	3
Directional axes	X, Y, Z

**Table 7:** Main modes of resonance discovered during the tests.

Form (Fashion)	Frequency (Hz)	Resonant Vibration Mode
1	350	Local vibration of the electronic unit—all 3 axes
2	550–580	Local vibration of the secondary mirror bracket—all 3 axes
3	630	Oscillations of the lower part of the payload along the main optical axis
4	730	Swinging of the secondary mirror bracket along the lateral axes
5	805	Oscillation of the electronics unit along the main optical axis
6	835–855	Swinging of the secondary mirror bracket along the lateral axes
7	1030–1090	Vibration of the entire structure—all 3 axes
8	1140–1180	Local vibration of the electronic unit—all 3 axes

**Table 8:** Resonance frequencies in the payload along the X-axis and comparison of shifts after each test.

Survey No.	Value	Modes						
		1	2	3	4	5	6	7+
1	Freq [Hz]	350	549	630	806	1056	1640	Peaks at 1800 Hz are characteristic of a sliding table with small oscillation amplitudes
2	Freq [Hz]	347	517	626	795	1039	1620	
	Shift [%]	0.9	5.8	0.6	1	1.6	1.2	
3	Freq [Hz]	349	520	626	795	1051	1630	
	Shift [%]	0.6	0.6	~0	~0	1.1	0.6	

**Table 9:** Resonance frequencies in the payload along the Y axis and comparison of shifts after each test.

Survey No.	Value	Modes							
		1	2	3	4	5	6	7	8+
1	Freq [Hz]	304	350.8	566	722.2	834	1087	1142	Peaks at 1800 Hz are characteristic of a sliding table with small oscillation amplitudes
2	Freq [Hz]	305	342	560	717.7	834	1087	1142	
	Shift [%]	0.3	2.5	1.1	0.6	~0	~0	~0	
3	Freq [Hz]	303	342	561	718	833	1084	1142	
	Shift [%]	0.7	~0	0.2	~0	0.1	0.3	~0	

**Table 10:** Resonance frequencies in the payload along the Z axis and comparison of shifts after each test.

Survey No.	Value	Modes									
		1	2	3	4	5	6	7	8	9	10+
1	Freq [Hz]	305	344	579	736	792	854	1028	1180	1339	Peaks at 1800 Hz are characteristic of a sliding table with small oscillation amplitudes
2	Freq [Hz]	301	337	482	742	792	856	1035	1180	1339	
	Shift [%]	1.3	2	0.6	0.2	~0	0.2	0.7	~0	~0	
3	Freq [Hz]	302	340	484	740	795	856	1014	1182	1341	
	Shift [%]	0.3	0.9	0.4	0.3	0.4	~0	2	0.2	0.1	

**Table 11:** Results of RMS acceleration increases under random vibrations.

Axis	Acceleration	M4	M5	M6	M7
X	Simulations, dB	7.674	5.080	5.118	9.665
	Tests, dB	5.204	4.137	4.588	10.413
Y	Simulations, dB	5.768	-		5.080
	Tests, dB	6.530	5.884		5.596
Z	Simulations, dB	5.759	-		5.367
	Tests, dB	6.747	3.435	3.205	5.044

**Table 12:** Experimental values of the damping coefficient in different parts of the payload based on the half-power bandwidth method.

Direction	Location	Res. Frequency (Hz)	Amplitude (g)	ζ (%)
X	M4	520	8.75	2.6
	M7	625	41	0.9
	M5, M6	1015	3.8	1.7
	M7	1055	15	2.5
Y	M7	340	8.56	3.3
	M4	717	9.2	1.3
	M4	830	12.32	2.2
Z	M7	340	8.07	4.8
	M4	730	7.66	2.4
	M4	792	7.18	1.1
	M4	857	12.4	1.7
	M7	1180	9.9	1.3

## Data Availability

The data presented in this study are available upon request from the corresponding authors.

## Abbreviations

The following abbreviations are used in this manuscript:

ERS	Earth remote sensing
FEM	Finite element modeling
RMS	Root mean square
PSD	Power spectral density
